# Association between Urinary-Extracellular-Vesicle-Enriched Proteome Dynamics and Oncological Outcomes Following Concurrent Chemoradiation in Locally Advanced Cervical Cancer

**DOI:** 10.34133/csbj.0196

**Published:** 2026-08-06

**Authors:** Chutimon Promthong, Raphatphorn Navakanitworakul, Kanokwan Obaun, Jitti Hanprasertpong, Ekasak Thiangphak, Kittinun Leetanaporn

**Affiliations:** ^1^ Translational Medicine Research Center, Faculty of Medicine, Prince of Songkla University, Songkhla 90110, Thailand.; ^2^ Department of Biomedical Sciences and Biomedical Engineering, Faculty of Medicine, Prince of Songkla University, Songkhla 90110, Thailand.; ^3^ Department of Research and Medical Innovation, Faculty of Medicine Vajira Hospital, Navamindradhiraj University, Bangkok 10300, Thailand.; ^4^ Division of Gynecologic Oncology, Department of Obstetrics and Gynecology, Faculty of Medicine, Prince of Songkla University, Songkhla 90110, Thailand.

## Abstract

Although concurrent chemoradiation (CCRT) is the standard of care for locally advanced cervical cancer, oncological outcomes remain suboptimal. Noninvasive monitoring of treatment responses could improve therapeutic management. This study investigated the temporal dynamics of extracellular vesicle (EV)-enriched urinary proteomes in patients with locally advanced cervical cancer undergoing CCRT to identify physiobiological shifts and proteins associated with treatment response and survival. Urine samples from 42 patients were collected longitudinally before, 1 month post-, and 3 months post-CCRT completion (126 samples). Urinary EVs were enriched using strong-anion-exchange magnetic beads. Proteomic profiling was performed using liquid chromatography–tandem mass spectrometry. Differential protein abundance and time-varying Cox regression analyses were used to correlate the proteomic signatures with CCRT response and overall survival. Analysis of longitudinal samples yielded 2,352 quantifiable proteins. Of these, 1,055 exhibited significant temporal shifts. Pathway analysis revealed that tumors and virus-associated proteins peaked at 3 months posttreatment, corresponding to the clinical response assessment window. By 3 months, nonresponders exhibited pronounced suppression of adaptive immune pathways and up-regulation of protein homeostasis and degradation pathways. Time-varying survival analysis identified 8 proteins driven by an altered T-complex ring complex/chaperonin containing T-complex protein 1, ribosomal proteins, and immune-related proteins associated with overall survival. Six of these were expressed in cervical tumor tissues based on the Human Protein Atlas. Overall, EV-enriched urinary proteomics provides a noninvasive liquid biopsy method for monitoring biological dynamics during CCRT. The observed immune suppression in nonresponders and the 8-protein prognostic signature offer preliminary insight into survival patterns and could help guide future studies on adjuvant immune-enhancing approaches.

## Introduction

Locally advanced cervical cancer (LACC) remains a challenging disease to eradicate because of tumor extension beyond operable limits. Currently, concurrent chemoradiation (CCRT) is the mainstay treatment [1]; however, oncological outcomes remain suboptimal [2,3]. One plausible explanation is the lack of understanding regarding the systematic biological responses throughout the course of treatment. After CCRT, the critical follow-up period is 1 and 3 months [1]. Characterizing the biological changes at these intervals may provide a deeper understanding of how the host responds to disease and thus improves therapeutic strategies.

Recent evidence suggests that urine may serve as an attractive source of biological markers reflecting disease-relevant processes even under nonurological conditions [4–6]. Urine has been applied for the screening and diagnosis of cervical cancer [7–9]. However, insights into urinary dynamics during CCRT and their impact on oncological outcomes remain limited.

Advances in mass spectrometry (MS)-based bottom-up proteomic techniques have substantially improved protein identification and quantification, enabling the detection of biologically relevant entities [10]. Nevertheless, crude proteins from urinary samples can be confounded by nonrelevant protein backgrounds. Extracellular vesicles (EVs) secreted by cells are considered an important source of biologically relevant signals, particularly in biomarker studies [4,11]. Accordingly, various EV-enrichment techniques have been developed [12,13]. Bead-based enrichment was recently introduced and has gained substantial traction for plasma samples owing to its efficiency in the enriching EVs and reducing background proteins [14,15]. Moreover, these approaches have demonstrated effective performance in urinary samples [16,17].

In this study, we performed time-series proteomic profiling of EV-enriched urine samples from patients with LACC who received CCRT to elucidate the temporal changes in the urinary proteome and their association with oncological outcomes, particularly treatment response and survival.

## Materials and Methods

### Patient cohorts and sample collection

All the study procedures were approved by the relevant Institutional Review Board (REC.68-445-38-1). We used leftover peripheral urine samples from patients with LACC who received CCRT treatment at Songklanagarind Hospital between 2021 October and 2024 January; the CCRT protocol has been described previously [18]. The eligibility criteria included a confirmed diagnosis of cervical cancer stage IB3-IVA based on the International Federation of Gynecology and Obstetrics (FIGO) 2018 criteria, no prior history of hysterectomy, and completion of the full course of CCRT. Patients with a second primary malignancy, a human immunodeficiency virus infection, or who were pregnant were excluded. The comprehensive CCRT protocol is detailed in Supplementary Methods.

Midstream urine samples were collected at 3 time points: before CCRT initiation (T1), 1 month post-CCRT completion (T2), and 3 months post-CCRT completion (T3). Collected urine was centrifuged at 2,500*g* for 15 min within 2 h of collection and stored at –80 °C until further analysis.

After treatment, patients followed a surveillance protocol involving follow-ups every 3 months in the first to second year, every 6 months in the third to fifth year, and annually thereafter. The initial CCRT response status was determined at T3 via pelvic examination by gynecologists, and the contrast-enhanced computed tomography results using the Response Evaluation Criteria in Solid Tumors (RECIST) [19] were evaluated by independent radiologists. Complete tumor absence from both clinical and imaging assessments was defined as a complete response (CR), whereas any residual tumor or new lesions were classified as a nonresponse (NR). Pathological confirmation was performed if the tumor was accessible.

### Enrichment of EVs and protein digestion

Urine EVs were enriched using MagResyn strong-anion-exchange (SAX) beads (ReSyn Bioscience, Edenvale, Gauteng, South Africa) following the Mag-Net protocol [14], with some modifications for urine. Briefly, 250 μl of urine was diluted with an equal amount of 100 mM bis–tris propane and 150 mM NaCl (pH 6.3) mixed with 25 μl of beads. Samples were incubated at room temperature with gentle shaking for 30 min. Thereafter, the beads were washed 3 times with 50 mM bis–tris propane + 150 mM NaCl. EV-enriched proteins were lysed with 2% sodium dodecyl sulfate in 50 mM tris-HCl and subsequently reduced and alkylated with 20 mM tris(2-carboxyethyl)phosphine + 10 mM chloroacetamide in a dark room at room temperature for 30 min. Proteins were precipitated with 70% acetonitrile (ACN) and washed twice with 70% ethanol and once with 100% ACN. On-bead digestion was performed using 2 μl of trypsin in 50 mM triethylammonium bicarbonate at 37 °C overnight. The digested peptides were quenched with 1% formic acid (FA) before being injected into the liquid chromatography–tandem MS (LC–MS/MS) system. Samples were processed in 6 batches of 21, with CR and NR samples evenly distributed across 2 batches per time point. The allocation was reversed between batches to ensure a balanced group representation throughout the acquisition run. For EV characterization, vesicles captured on beads from 2 representative samples were allocated for nanoparticle tracking analysis and transmission electron microscopy, with detailed protocols provided in Supplementary Methods.

### LC–MS/MS analysis

Analysis of peptides were performed using a Vanquish Neo ultrahigh-performance liquid chromatography system operating at a flow rate of 0.3 μl/min, coupled with an Orbitrap Exploris 480 (Thermo Fisher Scientific, Waltham, MA, USA) via an EASY-spray source. The peptides were injected into an Acclaim PepMap C18 trap column (3 μm, 75 μm × 2 cm) and separated on a PepMap C18 analytical column (2 μm, 75 μm × 500 mm). Gradient elution was performed linearly from 0% to 45% B over 90 min at 60 °C, with the following composition: 100% H_2_O + 0.1% FA as A and 80% ACN/20% H_2_O + 0.1% FA as B. The eluted peptides were analyzed in a data-independent manner using described parameters: MS1: 400 to 1,000 mass-to-charge ratio at a resolution of 120,000 and automatic gain control target of 300%; MS2: 20 mass-to-charge ratio isolation windows at a resolution of 30,000 with optimized window placement, automatic gain control target of 2,000%, and normalized collision energy of 28% with higher-energy collisional dissociation.

### Raw file processing

The raw spectra were searched against the UniProt *Homo sapiens* proteome (UP000005640) using DIA-NN version 2.5.0 [20], with the following parameters: one missed cleavage, N-terminal excision, carbamidomethylation as a fixed modification, generic scoring, 7 parts per million (ppm) of MS1 and 10 ppm of MS2 mass accuracies, QuantUMS quantification, retention-time-dependent normalization, and match-between-runs. Precursor and protein identification were filtered using a 1% false discovery rate.

### Statistical analysis

Clinicopathological characteristics are presented as median (Q1 to Q3) for continuous variables and numbers with percentages for categorical variables. Between-group comparisons were performed using the Wilcoxon rank sum test for continuous variables and Fisher’s exact test or Pearson’s chi-squared test for categorical variables, with statistical significance set at *P* < 0.05.

For proteomics, EV-related protein data were obtained from ExoCarta [21] and minimal information for studies of extracellular vesicles (MISEV) 2023 markers [22]. Protein-level quantification was conducted using the limpa R package, which log-transforms peptide intensities and estimates protein abundance from peptide-level data via a detection probability curve for label-free quantification [23,24]. Prior to statistical analysis, only the proteins detected in >50% of the samples from at least one study group were retained, followed by median normalization. To assess batch effects, samples were assigned to 6 acquisition batches (21 samples per batch). Multidimensional scaling (MDS) plots incorporating standard errors, principal components analysis (PCA), and quantitative variance partitioning were performed on the log_2_-transformed protein intensities before and after median normalization. MDS and PCA plots were colored by batch, time point, and response group. Variance partitioning decomposed each protein’s expression variance into components attributable to batch, patient, response status, and time point. Protein-level differential abundance analysis was conducted using a Bayesian moderated linear model in limma as part of the limpa workflow, using the patient as a blocking factor to account for repeated measures within the same patients. Significant protein changes over time were determined using the moderated *F* test, while differences between 2 groups were evaluated using the moderated *t* test with stage as covariate, with the significance threshold set at a fold change difference of ≥1.5 and *q* < 0.05.

For survival analysis, urine samples were collected at 3 time points: T1 (pretreatment), T2 (1 month after end of treatment), and T3 (3 months after end of treatment). Protein measurements at these time points were using time-varying Cox proportional hazards model. Each patient contributed up to 3 intervals: [0, *t*_2_), [*t*_2_, *t*_3_), and [*t*_3_, follow-up end), where *t*_2_ and *t*_3_ were defined as 1 and 3 months after the end of radiotherapy, respectively, based on individual treatment durations. At each interval, the protein expression value measured at the start of that interval was used. Models were fitted with protein expression as the primary predictor, cancer stage as a covariate, and a robust sandwich variance estimator applied to account for the correlation of repeated measurements within the same patient, with a significance threshold of *q* < 0.05. Overall survival (OS) was defined as the time from treatment initiation to death from any cause. Patients without such events were censored at the date of their last follow-up. We also performed a bootstrap analysis to confirm the robustness of the results (detailed in Supplementary Methods).

Functional annotation and pathway enrichment analyses were performed using overrepresentation analysis or gene set enrichment analysis (GSEA; Benjamini–Hochberg-adjusted *P* < 0.05) across multiple databases: JensenLab COMPARTMENTS (enrichR) for EV subcellular localization, Gene Ontology (GO) biological process (BP) per cluster (clusterProfiler/ClusterGVis) for time-dynamic proteins, and GO BP GSEA ranked by log_2_ fold change × *q* value with semantic simplification (cutoff = 0.5) for the response signature. Protein–protein interaction network analysis and Reactome pathway enrichment of prognostic proteins were performed using the STRING web tool (string-db.org, version 12.0). Data were visualized using ggplot2. All analyses were conducted using R version 4.5.2 (R Foundation for Statistical Computing, Vienna, Austria), unless stated otherwise.

For each candidate protein identified in the differential expression and survival analyses, expression in cervical cancer tissue was annotated using the Human Protein Atlas [25]. The cancer pathology dataset was downloaded and filtered for cervical cancer entries, and immunohistochemical staining intensity was classified as high, medium, low, or not detected on the basis of the highest staining level reported across tumor samples. Proteins without available antibody data were recorded as “no antibody”.

## Results

### Clinicopathologic characteristics of study cohort

**Fig. 1.**
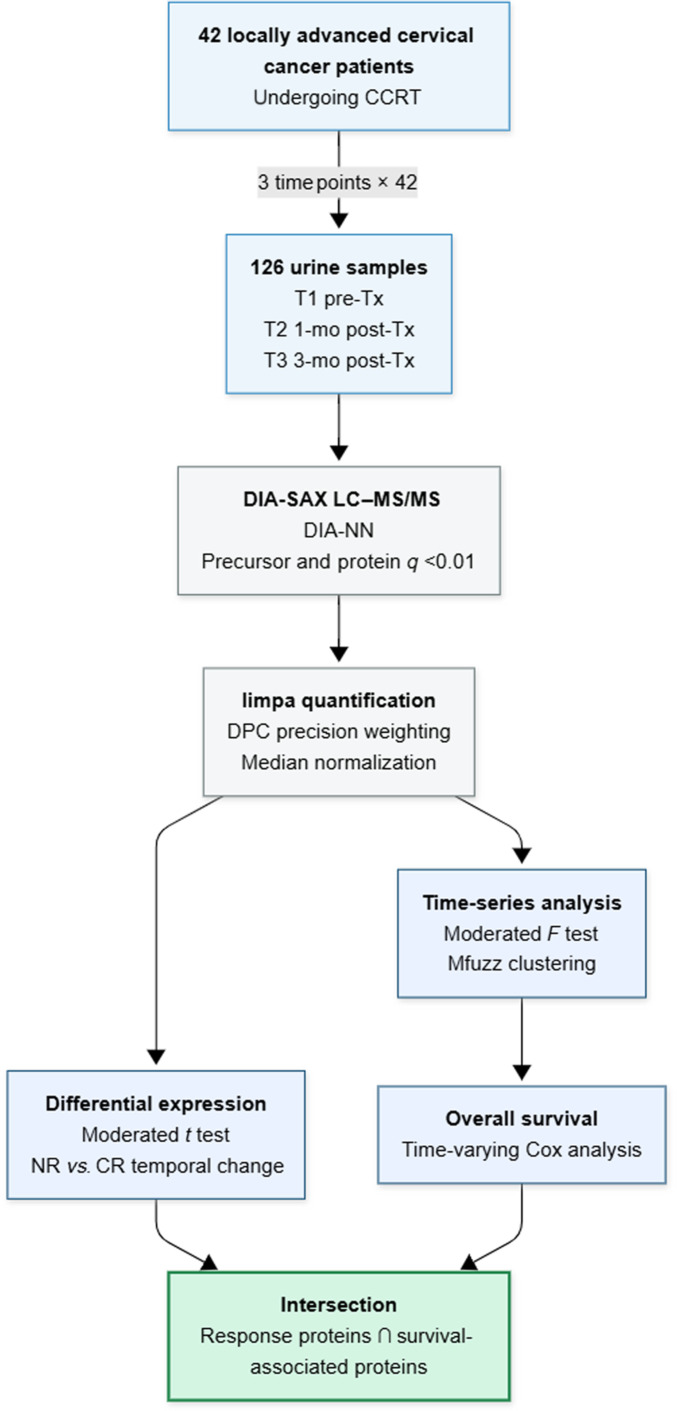
Study workflow. DIA, data-independent acquisition; DPC, detection probability curve; Tx, treatment.

The overall workflow is presented in Fig. 1. Forty-two patients were included in this analysis. The clinicopathological characteristics of the patients are shown in Table 1. The median age was 47 years (Q1 to Q3, 39.0 to 54.0 years). There were no significant associations between response status and age, body mass index, histologic subtype, or baseline laboratory parameters. In contrast, patients with stage IIIC/IVA disease were more likely to be nonresponders to CCRT (77.8% *vs.* 22.2%, *P* = 0.037).

**Table 1.** Baseline clinical characteristics of patients stratified by treatment response

Characteristic	Overall	CR	NR	*P* value^b^
	*N* = 42^a^	*n* = 24^a^	*n* = 18^a^	
**Age (years)**	47.0 (39.0–54.0)	48.5 (41.5–54.5)	43.0 (38.0–50.0)	0.154
**BMI category**				>0.999
Normal/underweight	19 (45.2%)	11 (45.8%)	8 (44.4%)	
Overweight	18 (42.9%)	10 (41.7%)	8 (44.4%)	
Obese	5 (11.9%)	3 (12.5%)	2 (11.1%)	
**FIGO stage**				**0.037**
IB3-IIIB	17 (40.5%)	13 (54.2%)	4 (22.2%)	
IIIC/IVA	25 (59.5%)	11 (45.8%)	14 (77.8%)	
**Histology**				0.258
SCC	33 (78.6%)	17 (70.8%)	16 (88.9%)	
ADC/ASC	9 (21.4%)	7 (29.2%)	2 (11.1%)	
**Hemoglobin (g/dl)**	11.45 (9.40–13.30)	12.20 (9.85–13.40)	11.25 (9.40–13.20)	0.492
**WBC (×10** ^**3**^ **/μl)**	7.70 (6.22–10.15)	7.88 (6.34–9.82)	7.39 (6.21–10.49)	>0.999
**Platelets (×10** ^**3**^ **/μl)**	321 (257–451)	325 (280–436)	303 (253–451)	0.520
**Plasma creatinine (mg/dl)**	0.64 (0.56–0.76)	0.64 (0.55–0.77)	0.64 (0.58–0.73)	0.629

^a^ Median (Q1 to Q3); *n* (%).

^b^ Wilcoxon rank sum test, Fisher’s exact test, and Pearson’s chi-square test.

CR, complete responder group; NR, nonresponder group; SCC, squamous cell carcinoma; ADC, adenocarcinoma; ASC, adenosquamous carcinoma. FIGO staging based on 2018 criteria; BMI, body mass index; WBC, white blood cell. The bold number indicates *P* < 0.05.

The median follow-up time was 43 months (Q1 to Q3, 32.3 to 48.5 months). The 3-year OS was 62.2% (95% confidence interval [CI], 48.6 to 79.6). Additional details regarding OS events, censoring, follow-up by group, and RECIST-defined clinical responses are presented in Tables S1 and S2.

### Characterization of proteins enriched from SAX-based digestion protocol

A total of 4,354 proteins were identified across 126 urinary samples derived from 42 patients. Of these, we retained peptides that were quantifiable in >50% of the samples within each group, resulting in 2,352 proteins (Tables S3 to S5) for downstream analysis. MDS and PCA plots demonstrated no systematic batch effects, with samples from all batches distributed uniformly across the component spaces. Furthermore, variance partitioning showed that the batch effect accounted for near-zero variance (Fig. S1).

**Fig. 2.**
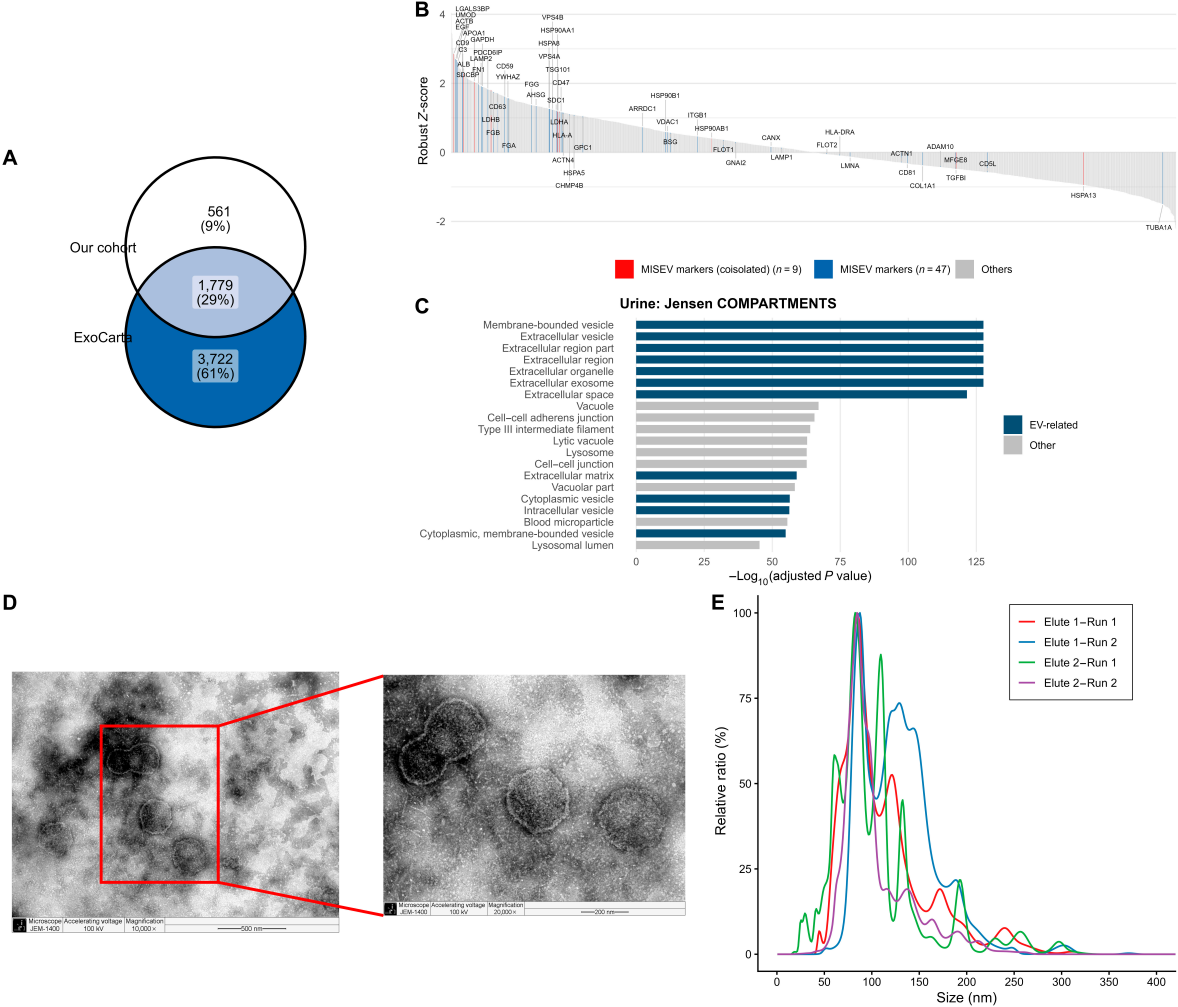
Characterization of the urine proteome as an extracellular vesicle (EV)-enriched compartment. (A) Overlap between proteins identified in the present cohort and the ExoCarta top 100 EV protein database. (B) All quantified proteins ranked by average log_2_ intensity (robust *z*-score), with MISEV guideline markers highlighted. (C) JensenLab COMPARTMENTS enrichment analysis of the detected proteome. (D) Transmission electron microscopy image of eluted, EV-enriched particles. (E) Particle size distribution determined by nanoparticle tracking analysis.

We confirmed EV enrichment of urinary preparations based on the median normalized total protein intensities across samples. Across the cohorts, 1,779 proteins (76.03%) overlapped with entries in the ExoCarta database (Fig. 2A). Robust *z*-scores of total protein intensities demonstrated that many proteins aligned with canonical MISEV markers (Fig. 2B and Table S6), including classical markers such as tetraspanins (CD9, CD63, and CD81), ALG-2-interacting protein X (PDCD6IP), and flotillin, with most of these exceeding the cohort median. As expected, some co-isolated proteins, namely, uromodulin and albumin, remained present, reflecting the highly abundant proteins that are commonly retained in urine. Further functional enrichment analysis confirmed that the majority of enriched proteins was EV-related (Fig. 2C). We also confirmed EV morphology by transmission electron microscopy, which demonstrated circular particles with a visible lipid bilayer (Fig. 2D). Nanoparticle tracking analysis showed a particle size distribution of approximately 50 to 200 nm, consistent with typical EV size ranges (Fig. 2E).

### Global time-dependent shifts in the urinary EV proteome

**Fig. 3.**
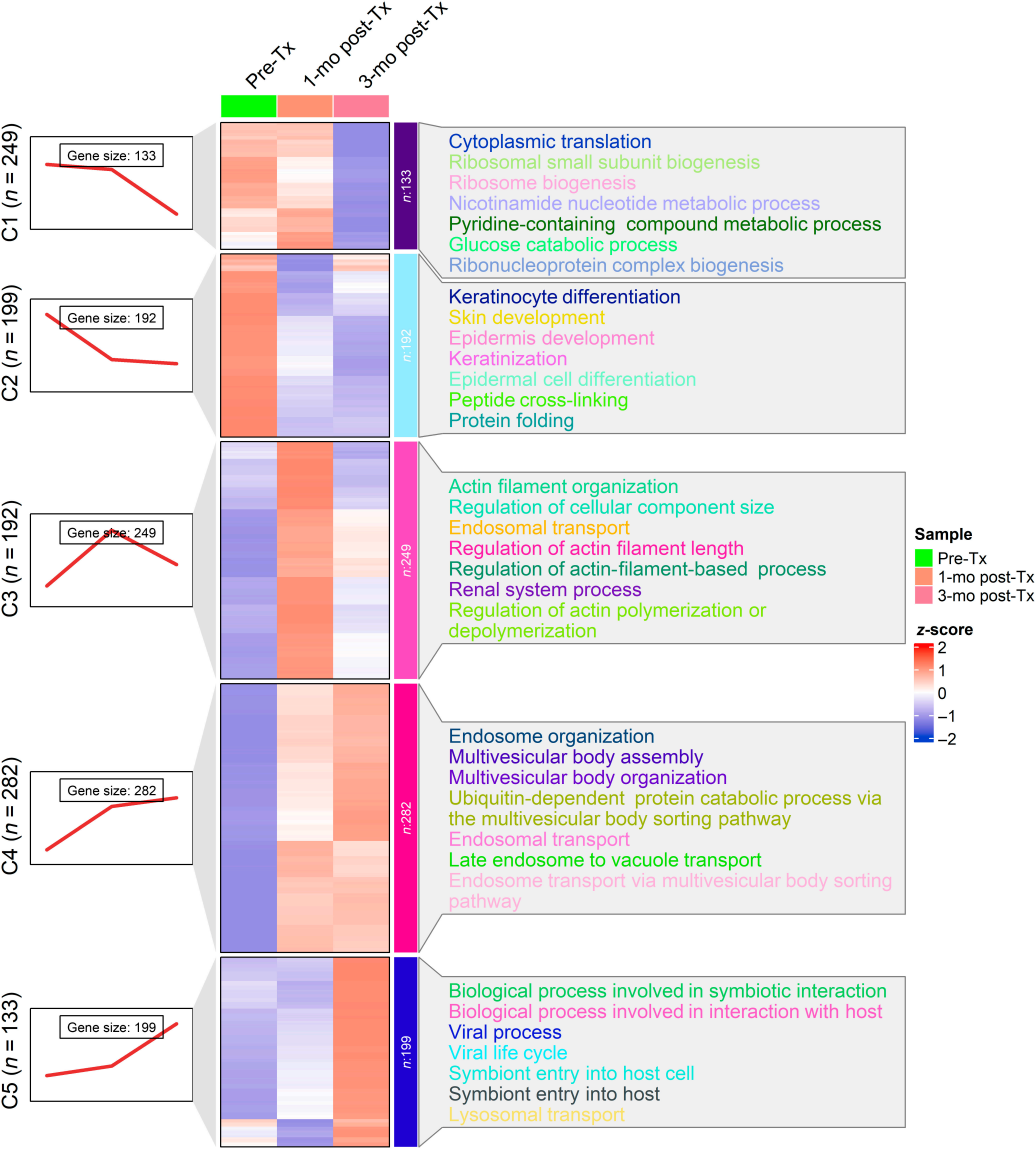
Time-dynamic urine proteome during concurrent chemoradiation (CCRT). Stage-adjusted protein expression was averaged per time point across all patients and soft-clustered (Mfuzz, *k* = 5). Heatmap rows represent significant proteins (any change across T1, T2, and T3; *q* < 0.05, analyzed using analysis of variance [ANOVA]); columns represent pretreatment (pre-Tx), 1-month post-Tx completion, and 3-month post-Tx completion. Top Gene Ontology biological process (GO BP) terms enriched per cluster are shown on the right.

We examined the impact of CCRT on urinary protein abundance across the 3 sampling time points (Fig. 3 and Fig. S2). Overall, 1,055 proteins showed significant changes at one or more time points (*q* < 0.05; Table S7). Unsupervised clustering partitioned the temporal profiles into distinct clusters. Cluster (C3) displayed a pronounced increase at T2, followed by a decrease at T3, and was mainly associated with endosomal transport, actin filament organization, and renal system processes.

Two additional clusters exhibited progressive increases. Cluster C4 markedly increased in abundance at T2 and peaked at T3, whereas cluster C5 exhibited the largest increase after T2. C4 was enriched for ubiquitin-dependent catabolic processes, whereas C5 was associated with pathways related to viral budding into vesicles, suggesting a coordinated regulation of these processes over the course of treatment. In contrast, clusters C1 and C2 exhibited a continuous decrease over time. C1 was associated with cytoplasmic translation and ribosome-related processes, whereas C2 was enriched in keratinocyte differentiation and protein folding.

### Differential protein abundance and the associated pathway enrichment between CCRT response groups

**Fig. 4.**
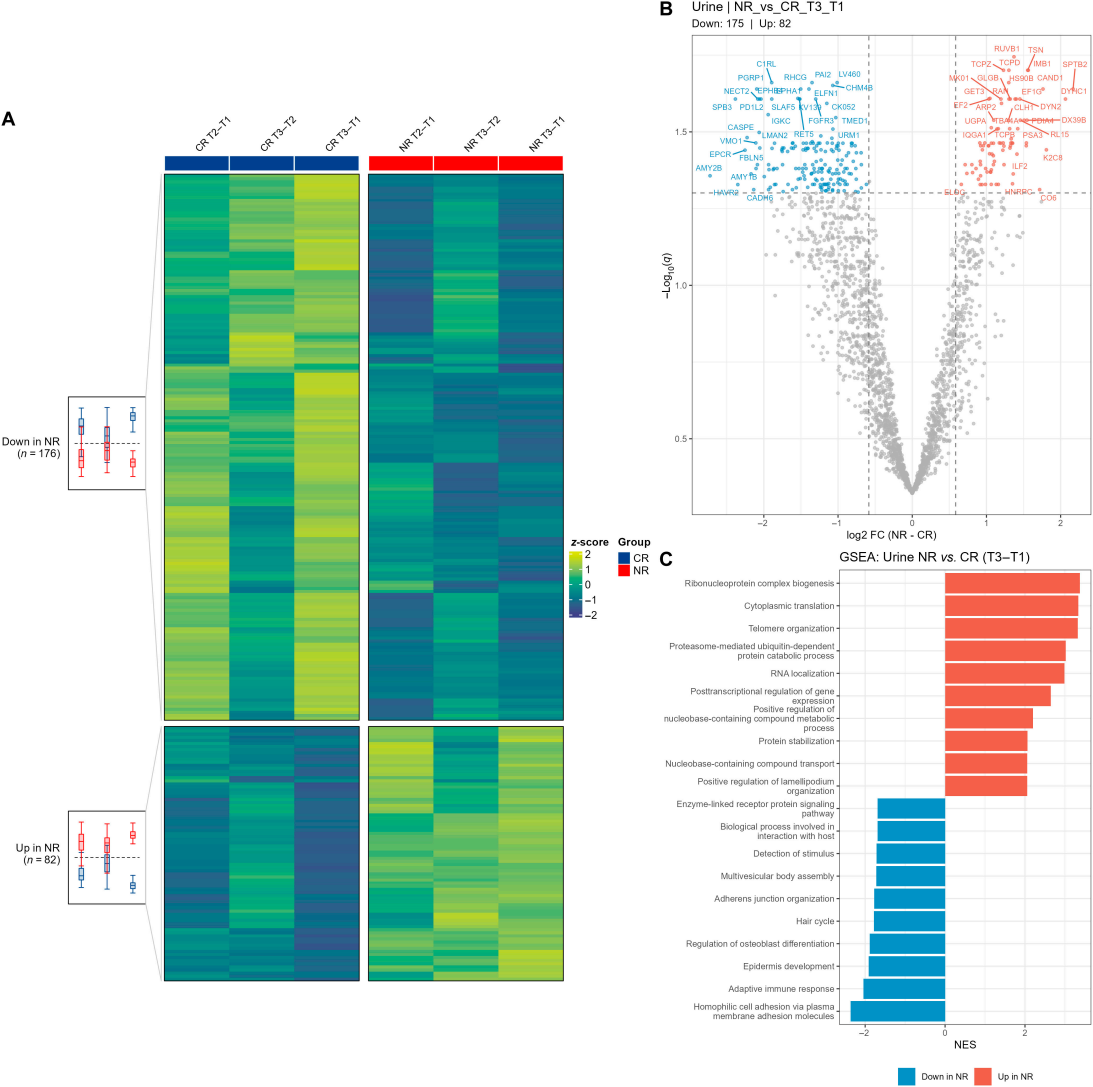
Differential dynamic response signature in urine. (A) Heatmap of proteins with significantly different temporal trajectories between nonresponse (NR) and complete response (CR) groups in at least one interval [T2−T1, T3−T2, or T3−T1; |log_2_ fold change| > log_2_(1.5), *q* < 0.05]. Columns show per-group mean log_2_ fold changes across intervals. (B) Volcano plot for NR *vs.* CR groups in T3−T1 contrast; dashed lines indicate |log_2_ fold change| > log_2_(1.5) and *q* < 0.05 thresholds. (C) gene set enrichment analysis (GSEA) (Gene Ontology biological process [GO BP]) ranked by NR *vs.* CR T3−T1 log_2_ fold change; top 10 positively and negatively enriched terms after semantic simplification (cutoff = 0.5) are shown. NES, normalized enrichment score.

Between the NR and CR groups, assessed using group-by-time contrasts [(NR_T*i*_ − NR_T*j*_) − (CR_T*i*_ − CR_T*j*_), where *i* and *j* denote the time points], no major difference in protein abundance was observed at T2–T1 or T3–T2, except for a single protein at T2–T1 (Fig. 4A, Fig. S3, and Table S8); however, a distinct difference was observed at T3–T1. In particular, 82 proteins were up-regulated, and 175 were down-regulated in the NR group (Fig. 4B). The GSEA revealed that up-regulated proteins were enriched in pathways related to telomere organization, proteasome mediation, and cell proliferation. In contrast, proteins that were significantly down-regulated were associated with cell adhesion and adaptive immune response and multivesicular body (Fig. 4C).

### Impact of urinary EV proteome on patient survival

**Fig. 5.**
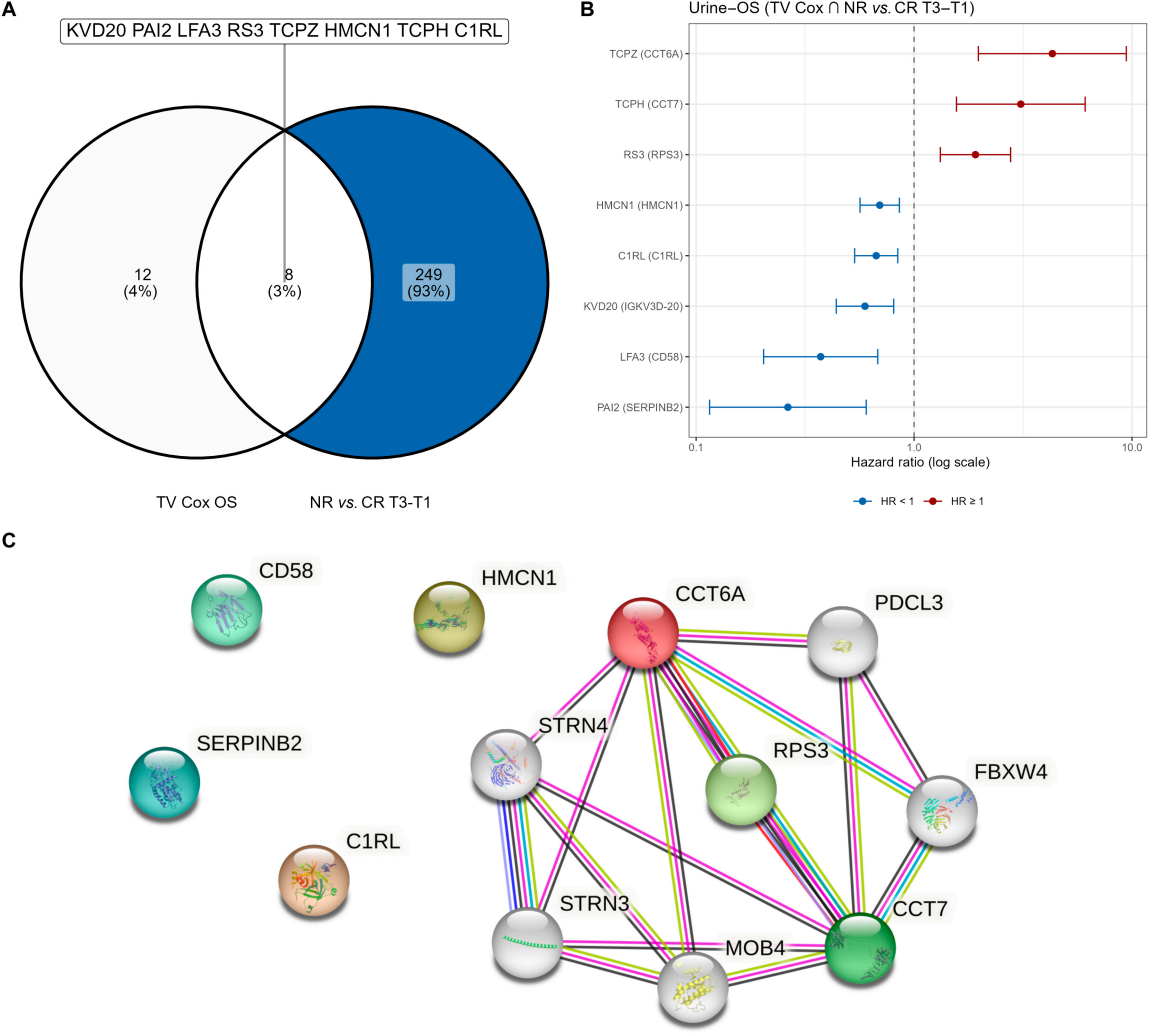
Urine proteins associated with overall survival (OS) identified by time-varying (TV) Cox regression. (A) Intersection of proteins significantly associated with OS and differentially abundant proteins between nonresponse (NR) and complete response (CR) at T3−T1. Gene names of the intersection proteins are annotated. (B) Forest plot of hazard ratios (HRs) for intersection proteins; points are colored by direction (red, HR ≥ 1; blue, HR < 1). Error bars indicate 95% confidence interval (CI). (C) Search Tool for the Retrieval of Interacting Genes/Proteins protein–protein interaction network of intersection proteins (predicted interacting proteins shown in white).

Focusing on proteins that exhibited a temporal shift during treatment, a time-varying Cox model without stratification by response status showed that, after adjusting for cancer stage, 20 proteins were associated with OS (Fig. S4 and Tables S9 and S10), of which 8 overlapped with proteins that were also dysregulated in the same direction as in nonresponders (Fig. 5A and B and Table 2).

**Table 2.** Summary of proteins differentially expressed in nonresponders with prognostic impact on overall survival and corresponding tissue immunostaining data from the Human Protein Atlas

Protein names	Gene	Log_2_FC NR *vs.* CR (95% CI)	HR OS (95% CI)	HPA staining
PAI2 (plasminogen activator inhibitor-2)	*SERPINB2*	−1.35 (−2.04 to −0.66)	0.26 (0.12 to 0.60)	High
LFA3 (lymphocyte function-associated antigen 3)	*CD58*	−1.18 (−2.06 to −0.30)	0.37 (0.20 to 0.68)	No antibody
KVD20 (human immunoglobulin kappa variable 3D-20)	*IGKV3D-20*	−1.75 (−2.90 to −0.60)	0.60 (0.44 to 0.81)	Not detected
C1RL (complement C1r subcomponent-like)	*C1RL*	−1.89 (−2.86 to −0.92)	0.67 (0.53 to 0.84)	High
HMCN1 (hemicentin 1)	*HMCN1*	−2.05 (−3.40 to −0.71)	0.70 (0.57 to 0.86)	Medium
RS3 (ribosomal protein S3)	*RPS3*	1.35 (0.50 to 2.21)	1.91 (1.32 to 2.77)	High
TCPH (T-complex protein 1 subunit eta)	*CCT7*	0.81 (0.23 to 1.40)	3.09 (1.56 to 6.09)	Medium
TCPZ (T-complex protein 1 subunit zeta)	*CCT6A*	1.23 (0.63 to 1.83)	4.31 (1.97 to 9.41)	Medium

Log_2_FC, log_2_ fold change; NR, nonresponder group; CR, complete responder group; HR, hazard ratio; OS, overall survival; CI, confidence interval; HPA, Human Protein Atlas.

Protein–protein interaction analysis of the overlapping proteins revealed that ribosomal protein S3 (RPS3), chaperonin containing TCP1 subunit 6A (CCT6A), and CCT7 formed a connected cluster, which further expanded upon the inclusion of predicted interactors (noncolor) to striatin family members, striatin-interacting phosphatase and kinase (STRIPAK) components (striatin 3 [STRN3], STRN4, MOB-like protein phocein-4 [MOB4], and F-box/WD repeat-containing protein 4 [FBXW4]), and the co-chaperone phosducin-like protein 3 (PDCL3) (Fig. 5C). In contrast, plasminogen activator inhibitor 2 (SERPINB2), hemicentin-1 (HMCN1), complement C1r subcomponent Like (C1RL), and cluster of differentiation 58 (CD58) appeared as isolated nodes, consistent with the more heterogeneous functions related to the extracellular matrix, complement/serine protease activity, and immune regulation.

To confirm the robustness of this finding, we performed a sensitivity analysis that included all detected proteins and obtained similar results (more proteins were identified with all temporal changes retained; Figs. S5 and S6 and Table S11). We assessed whether the dysregulated proteins were concomitantly present in cervical tissue by cross-referencing them with the Human Protein Atlas immunohistochemistry database. Six of the 8 proteins (excluding lymphocyte function-associated antigen 3 [LFA3] and human immunoglobulin kappa variable 3D-20 [KVD20]) showed detectable staining in cervical cancer tissue (Table 2), indicating that most urinary proteins associated with poor outcomes were also expressed in cervical tumors. In addition, we performed patient-level bootstrap resampling (*n* = 1,000). All 8 proteins retained statistical significance, with a consistent direction of effect in the majority of resamples, ranging from 63.4% (C1RL) to 85.5% (CCT6A) (Table S12). Overall, these observations suggest that the identified urinary proteins may be biologically related to cervical cancer, although conclusions cannot be drawn about the precise cellular origin of the urinary EVs from these data alone.

## Discussion

In the time-course analysis, the trends in urinary proteomes changed over the course of CCRT. Notably, there were significant downward trends in proteins related to cell activation, metabolic reprogramming, and survivability, corresponding to a reduced tumor burden after CCRT. In contrast, viral and host–virus interaction proteins peaked at 3 months posttreatment. This may reflect viral protein interactions within the tumor microenvironment, where human papillomavirus persists in a latent state while evading immune surveillance [26]. Radiation induces necrosis or apoptosis of cancerous cells, releasing tumor-associated proteins into the circulation [27]. Concurrently, we observed the global up-regulation of protein ubiquitination and several EV-associated pathways, including the endosomal sorting complexes required for transport machinery and multivesicular body organization, which was substantially less pronounced in nonresponder. Collectively, these findings suggest that tumor-associated proteins were maximally detected in the urine 3 months after treatment, likely shed via EV-mediated mechanisms, and that this process is impaired in nonresponders. Remarkably, this timeline coincides with the 3-month time point recommended for clinical response assessment following CCRT [28].

Although radiation is effective in eradicating tumors, it impairs adaptive immune activation and alters metabolic reprogramming [29,30]. In this context, we observed that the NR group exhibited greater suppression of the adaptive immune response at the pathway level as early as 1 month post-CCRT completion, which became more pronounced at 3 months. Previous studies demonstrated that adaptive immune-enhanced strategies, such as tumor infiltration therapy [31] and programmed cell death ligand 1 inhibitor treatment [32,33], may improve treatment efficacy in cervical cancer. Our findings support the potential efficacy of such strategies, particularly in restoring inadequate adaptive immune responses, which are more severely suppressed in the NR group.

Time-varying analysis identified several proteins as prognostic markers, 8 of which also showed dysregulation after CCRT. Notably, a subset of proteins were members of the T-complex protein ring complex/chaperonin containing T-complex protein 1 (TRiC/CCT) families. As critical regulators of cytoskeleton assembly, TRiC/CCT proteins are frequently overexpressed and associated with poor clinical outcomes in multiple cancer types [34–38]. Studies in cancer models have demonstrated that CCT proteins driven by oncogenic alterations establish an immunosuppressive tumor microenvironment and redirects transforming growth factor-β signaling toward its prometastatic arm [38,39]. In addition, we observed the dysregulation of the 40*S* ribosomal protein RPS3, which constitutively activates nuclear factor κB, thereby enhancing tumor progression and survival [40]. Finally, several immunoglobulin and complement proteins were altered as largely standalone nodes, which may reflect impaired or reshaped immune responses in patients with poorer survival.

In this study, we used SAX beads for EV enrichment, which relies on electrostatic interactions between positively charged quaternary ammonium groups [14]. Our results are consistent with those of previous reports that showed effective enrichment in various biofluids [14,16,41]. However, we did not use this strategy for “isolation” or “purification” as it does not yield purely isolated specific urinary EV subpopulations but rather captures a heterogenous EV-enriched fraction.

To the best of our knowledge, this is the first study to investigate the temporal effect of CCRT on urinary EV proteomes in LACC. Although the tumor tissue remains the most informative matrix, tissue sampling in LACC is frequently limited by poor tumor accessibility and extensive tumor necrosis. In contrast, urine can be collected noninvasively and repeatedly, providing a practical matrix for longitudinal monitoring. Our study found that 6 of 8 effector proteins were detected in cervical tissue, suggesting that urinary proteomes are relevant to cervical cancer biology. However, this should be interpreted with caution and not as direct evidence that the urinary vesicles themselves originate from cervical tumors. In addition, while our previous work required a combination of multiple plasma EV proteins in a multivariate model to predict CCRT response status [42], the present study found that the corresponding signature in urinary EVs is more pronounced and associated with survival. Experimental data from mouse models demonstrated that tumor-derived EVs can cross the glomerulus and, notably, become more concentrated in urine than in plasma [6].

This study has several limitations. First, the sample size was modest, the study was conducted at a single center, and matched tumor tissue was unavailable; therefore, the survival-associated signature and its direct link to paired tissue biology remain to be explored. Second, urine-specific confounders, such as hydration status, renal function, urinary tract inflammation, and diurnal variation, may influence EV protein profiles. Accordingly, although residual variability cannot be fully excluded, we implemented several measures to minimize these effects, including bead-based EV enrichment to standardize matrix complexity; median normalization, which efficiently correct global intensity differences across samples [43]; and the restriction of analyses to consistently detected proteins. Third, although the FIGO stage was included as a covariate, residual confounding by stage-correlated factors such as tumor volume and nodal burden cannot be excluded. Ultimately, these associations should be regarded as hypothesis-generating rather than immediately translatable, serving as a basis for validation in larger cohorts with orthogonal modalities that have more practical turnaround times for routine clinical use, such as targeted proteomics approaches or immunoassays. In addition, future studies incorporating clinical data, digital pathology results [44], and transcriptomic data [45], combined with artificial intelligence frameworks [46], may enable more accurate translation into personalized CCRT strategies for patients with LACC.

In conclusion, our findings suggest that the longitudinal profiling of EV-enriched urine can capture treatment-related changes in LACC. Our time-course analysis revealed that tumor and viral clearance peak at 3 months post-CCRT completion, consistent with the standard clinical assessment windows. Furthermore, we identified 8 proteins associated with both treatment response and survival in nonresponders. Taken together, our results suggest that urinary proteomics may serve as a noninvasive tool for monitoring tumor dynamics, although this hypothesis requires validation in larger cohorts with paired tissue samples.

## Ethical approval

All patients provided written informed consent before participating in the study, which involved the use of leftover specimens. Informed consent was obtained from all participants in accordance with the principles of the Declaration of Helsinki. Approval was obtained from the Ethics Committee of the Faculty of Medicine, Prince of Songkla University (REC.68-445-38-1).

## Data Availability

The raw MS/MS data and analysis files were deposited in the ProteomeXchange Consortium via the jPOST partner repository with dataset identifiers (PXD078395) and (JPST004627).

## References

[1] National Comprehensive Cancer Network. Cervical cancer. version 3.2024. National Comprehensive Cancer Network. May 2024. https://www.nccn.org/professionals/physician_gls/pdf/cervical.pdf

[2] Kim TE, Park BJ, Kwack HS, Kwon JY, Kim JH, Yoon SC. Outcomes and prognostic factors of cervical cancer after concurrent chemoradiation. J Obstet Gynaecol Res. 2012;38(11):1315–1320. 22612778 10.1111/j.1447-0756.2012.01871.x

[3] Liu J, Tang G, Zhou Q, Kuang W. Outcomes and prognostic factors in patients with locally advanced cervical cancer treated with concurrent chemoradiotherapy. Radiat Oncol. 2022;17(1):142. 35978412 10.1186/s13014-022-02115-1 PMC9386993

[4] Wang S, Kojima K, Mobley JA, West AB. Proteomic analysis of urinary extracellular vesicles reveal biomarkers for neurologic disease. EBioMedicine. 2019;45:351–361. 31229437 10.1016/j.ebiom.2019.06.021 PMC6642358

[5] Jain S, Lin SY, Song W, Su YH. Urine-based liquid biopsy for nonurological cancers. Genet Test Mol Biomarkers. 2019;23(4):277–283. 30986103 10.1089/gtmb.2018.0189 PMC6482900

[6] Kawaguchi S, Ajiri T, Mitsuya R, Tsuchiya R, Kunitake K, Tanaka Y, Yasui T. Glomerular routing of tumor-derived extracellular vesicles substantiates urinary biopsy. Sci Adv. 2026;12(8):eaeb0555. 41719393 10.1126/sciadv.aeb0555 PMC12922736

[7] Sahasrabuddhe VV, Gravitt PE, Dunn ST, Robbins D, Brown D, Allen RA, Wentzensen N. Evaluation of clinical performance of a novel urine-based HPV detection assay among women attending a colposcopy clinic. J Clin Virol. 2014;60(4):414–417. 24881489 10.1016/j.jcv.2014.04.016 PMC4146457

[8] Chokchaichamnankit D, Watcharatanyatip K, Subhasitanont P, Weeraphan C, Keeratichamroen S, Sritana N, Srisomsap C. Urinary biomarkers for the diagnosis of cervical cancer by quantitative label-free mass spectrometry analysis. Oncol Lett. 2019;17(6):5453–5468. 31186765 10.3892/ol.2019.10227 PMC6507435

[9] Du H, Dai W, Wu D, Liu Y, Xiao A, Huo J, Wu R. Expanding access to cervical cancer screening: The performance of self-sampled urine HPV testing. Int J Infect Dis. 2025;161: Article 108175. 41176152 10.1016/j.ijid.2025.108175

[10] Movassaghi CS, Sun J, Jiang Y, Turner N, Chang V, Chung N, Meyer JG. Recent advances in mass spectrometry-based bottom-up proteomics. Anal Chem. 2025;97(9):4728–4749. 40000226 10.1021/acs.analchem.4c06750 PMC12117541

[11] Greening DW, Xu R, Rai A, Suwakulsiri W, Chen M, Simpson RJ. Clinical relevance of extracellular vesicles in cancer—Therapeutic and diagnostic potential. Nat Rev Clin Oncol. 2025;22(12):924–952. 41062719 10.1038/s41571-025-01074-2

[12] Barreiro K, Dwivedi OP, Leparc G, Rolser M, Delic D, Forsblom C, Holthofer H. Comparison of urinary extracellular vesicle isolation methods for transcriptomic biomarker research in diabetic kidney disease. J Extracell Vesicles. 2020;10(2): Article e12038. 33437407 10.1002/jev2.12038 PMC7789228

[13] Teixeira-Marques A, Monteiro-Reis S, Montezuma D, Lourenço C, Oliveira MC, Constâncio V, Jerónimo C. Improved recovery of urinary small extracellular vesicles by differential ultracentrifugation. Sci Rep. 2024;14(1):12267. 38806574 10.1038/s41598-024-62783-9 PMC11133306

[14] Wu CC, Tsantilas KA, Park J, Plubell D, Sanders JA, Naicker P, MacCoss MJ. Enrichment of extracellular vesicles using mag-net for the analysis of the plasma proteome. Nat Commun. 2025;16(1):5447. 40595564 10.1038/s41467-025-60595-7 PMC12219689

[15] Iliuk A, Wu X, Li L, Sun J, Hadisurya M, Boris RS, Tao WA. Plasma-derived extracellular vesicle phosphoproteomics through chemical affinity purification. J Proteome Res. 2020;19(7):2563–2574. 32396726 10.1021/acs.jproteome.0c00151 PMC7479851

[16] Kuo SFT, Spall S, Emery-Corbin SI, Mohamed A, Dite T, Chow K, Webb AI. Quantitative proteomic analysis unveils protein concentration effects in neat urine compared to urine extracellular vesicles. J Proteome Res. 2025;24(7):3343–3355. 40512035 10.1021/acs.jproteome.5c00060

[17] Liu YK, Wu X, Hadisurya M, Li L, Kaimakliotis H, Iliuk A, Tao WA. One-pot analytical pipeline for efficient and sensitive proteomic analysis of extracellular vesicles. J Proteome Res. 2023;22(10):3301–3310. 37702715 10.1021/acs.jproteome.3c00361 PMC10897859

[18] Leetanaporn K, Hanprasertpong J. Predictive value of the hemoglobin-albumin-lymphocyte-platelet (HALP) index on the oncological outcomes of locally advanced cervical cancer patients. Cancer Manag Res. 2022;14:1961–1972. 35726336 10.2147/CMAR.S365612 PMC9206525

[19] Schwartz LH, Litière S, De Vries E, Ford R, Gwyther S, Mandrekar S, Seymour L. RECIST 1.1—Update and clarification: From the RECIST committee. Eur J Cancer. 2016;62:132–137. 27189322 10.1016/j.ejca.2016.03.081 PMC5737828

[20] Demichev V, Messner CB, Vernardis SI, Lilley KS, Ralser M. DIA-NN: Neural networks and interference correction enable deep proteome coverage in high throughput. Nat Methods. 2020;17(1):41–44. 31768060 10.1038/s41592-019-0638-x PMC6949130

[21] Mathivanan S, Simpson RJ. ExoCarta: A compendium of exosomal proteins and RNA. Proteomics. 2009;9(21):4997–5000. 19810033 10.1002/pmic.200900351

[22] Welsh JA, Goberdhan DCI, O’Driscoll L, Buzas EI, Blenkiron C, Bussolati B, Benedikter BJ. Minimal information for studies of extracellular vesicles (MISEV2023): From basic to advanced approaches. J Extracell Vesicles. 2024;13(2): Article e12404. 38326288 10.1002/jev2.12404 PMC10850029

[23] Li M, Smyth GK. Neither random nor censored: Estimating intensity-dependent probabilities for missing values in label-free proteomics. Bioinformatics. 2023;39(5):btad200. 37067487 10.1093/bioinformatics/btad200 PMC10174703

[24] Li M, Cobbold SA, Smyth GK. Quantification and differential analysis of mass spectrometry proteomics data with probabilistic recovery of information from missing values. BioRxiv. 2025. 10.1101/2025.04.28.651125

[25] Uhlén M, Fagerberg L, Hallström BM, Lindskog C, Oksvold P, Mardinoglu A, Pontén F. Tissue-based map of the human proteome. Science. 2015;347(6220):1260419. 25613900 10.1126/science.1260419

[26] Mangino G, Vincenza Chiantore M, Iuliano M, Capriotti L, Accardi L, Di Bonito P, Romeo G. Role of extracellular vesicles in human papillomavirus-induced tumorigenesis. In: Saxena KS, editor. Current perspectives in human papillomavirus. London (UK): IntechOpen; 2019. p. 137–160.

[27] Huang Y, Zou D, Guo M, He M, He H, Li X, Mao Z. HPV and radiosensitivity of cervical cancer: A narrative review. Ann Transl Med. 2022;10(24):1405. 36660629 10.21037/atm-22-5930 PMC9843372

[28] Abu-Rustum NR, Campos SM, Amarnath S, Arend R, Barber E, Bradley K, Sambandam V. Cervical cancer, version 2.2026, NCCN clinical practice guidelines in oncology. J Natl Compr Cancer Netw. 2025;23(12):549–573. 10.6004/jnccn.2025.0057 41671440

[29] Li HH, Yw W, Chen R, Zhou B, Ashwell JD, Fornace AJ. Ionizing radiation impairs T cell activation by affecting metabolic reprogramming. Int J Biol Sci. 2015;11(7):726–736. 26078715 10.7150/ijbs.12009 PMC4466454

[30] van Meir H, Nout RA, Welters MJP, Loof NM, de Kam ML, Van Ham JJ, van der Burg SH. Impact of (chemo)radiotherapy on immune cell composition and function in cervical cancer patients. Onco Targets Ther. 2016;6(2): Article e1267095. 10.1080/2162402X.2016.1267095 PMC5353924 28344877

[31] Zhu Y, Zhou J, Zhu L, Hu W, Liu B, Xie L. Adoptive tumor infiltrating lymphocytes cell therapy for cervical cancer. Hum Vaccin Immunother. 2022;18(5):2060019. 35468048 10.1080/21645515.2022.2060019 PMC9897649

[32] Colombo N, Dubot C, Lorusso D, Caceres MV, Hasegawa K, Shapira-Frommer R, Monk BJ. Pembrolizumab for persistent, recurrent, or metastatic cervical cancer. N Engl J Med. 2021;385(20):1856–1867. 34534429 10.1056/NEJMoa2112435

[33] Luo D, Yu Y, Wang Q, Peng T, Li C, Zhang W, Huang J. The benefit and risk of addition of PD-1/PD-L1 inhibitors to chemotherapy for advanced cervical cancer: A phase 3 randomized controlled trials based meta-analysis. BMC Cancer. 2025;25(1):450. 40075324 10.1186/s12885-025-13843-4 PMC11905652

[34] Huang K, Zeng Y, Xie Y, Huang L, Wu Y. Bioinformatics analysis of the prognostic value of CCT6A and associated signalling pathways in breast cancer. Mol Med Rep. 2019;19(5):4344–4352. 30942452 10.3892/mmr.2019.10100 PMC6472137

[35] Ma J, Yang L, Feng H, Zheng L, Meng H, Li X. CCT6A may act as a potential biomarker reflecting tumor size, lymphatic metastasis, FIGO stage, and prognosis in cervical cancer patients. J Clin Lab Anal. 2021;35(8): Article e23793. 34196992 10.1002/jcla.23793 PMC8373327

[36] Ma J, Wei Q, Zhang L, Sun F, Li W, Du R, Wang C. CCT6A functions as promising diagnostic biomarker and promotes cell proliferation in colorectal cancer. J Cancer. 2024;15(18):5897–5909. 39440061 10.7150/jca.98901 PMC11493007

[37] Liu H, Chen L, Chen Y, Jin Y, Chen X, Ma N, Chen Y. TCP1 promotes the progression of malignant tumours by stabilizing c-Myc through the AKT/GSK-3β and ERK signalling pathways. Commun Biol. 2025;8(1):563. 40185866 10.1038/s42003-025-07867-6 PMC11971430

[38] Deng L, Ruan J, Li Y. Exploring the role of CCT7 in prognosis, cell cycle regulation, and immune microenvironment remodeling of lung adenocarcinoma based on multi-omics datasets and functional experiments. Biochim Biophys Acta Gen Subj. 2026;1870: Article 130896. 41429196 10.1016/j.bbagen.2025.130896

[39] Ying Z, Tian H, Li Y, Lian R, Li W, Wu S, Li M. CCT6A suppresses SMAD2 and promotes prometastatic TGF-β signaling. J Clin Invest. 2017;127(5):1725–1740. 28375158 10.1172/JCI90439 PMC5409794

[40] Kim TS, Yi YW, Kim DJ, Seong YS. A review on ribosomal protein S3 (RPS3): Roles in cancer and its resistance to drugs. Int J Biol Macromol. 2025;318: Article 144955. 40480568 10.1016/j.ijbiomac.2025.144955

[41] Aastha A, De Macedo Filho LJM, Woolman M, Ignatchenko V, Keszei A, Remite-Berthet G, Mansouri A, Kislinger T. Comparative evaluation of analytical methods for CSF proteomics. Clin Proteomics. 2025;22(1):46. 41315961 10.1186/s12014-025-09568-y PMC12661759

[42] Leetanaporn K, Chiangjong W, Roytrakul S, Molika P, Janmunee N, Atjimakul T, Navakanitworakul R. Enhancing outcome prediction of concurrent chemoradiation treatment in patients with locally advanced cervical cancer through plasma extracellular vesicle proteomics. Heliyon. 2024;10(16): Article e36374. 39262965 10.1016/j.heliyon.2024.e36374 PMC11388600

[43] Zelter A, Riffle M, Merrihew GE, Mutawe B, Maurais A, Yang HY, Inman JL, Celniker SE, Mao JH, Wan KH, *et al*. Is protein quantification and physical normalization always necessary in proteomics? bioRxiv. 2026. 10.64898/2026.02.13.705808

[44] Wang YL, Gao S, Xiao Q, Li C, Grzegorzek M, Zhang YY, Li XH, Kang Y, Liu FH, Huang DH, et al. Role of artificial intelligence in digital pathology for gynecological cancers. Comput Struct Biotechnol J. 2024;24:205–212. 10.1016/j.csbj.2024.03.007 PMC10951449 38510535

[45] Fang J, Wang Y, Li C, Liu W, Wang W, Wu X, Wang Y, Zhang S, Zhang J. A hypoxia-derived gene signature to suggest cisplatin-based therapeutic responses in patients with cervical cancer. Comput Struct Biotechnol J. 2024;23:2565–2579. 38983650 10.1016/j.csbj.2024.06.007 PMC11231957

[46] Gumilar KE, Indraprasta BR, Faridzi AS, Wibowo BM, Herlambang A, Rahestyningtyas E, Irawan B, Tambunan Z, Bustomi AF, Brahmantara BN, et al. Assessment of large language models (LLMs) in decision-making support for gynecologic oncology. Comput Struct Biotechnol J. 2024;23:4019–4026. 10.1016/j.csbj.2024.10.050 PMC11603009 39610903

