# Supplementary material for: Association between Urinary-Extracellular-Vesicle-Enriched Proteome Dynamics and Oncological Outcomes Following Concurrent Chemoradiation in Locally Advanced Cervical Cancer
Source: Comput Struct Biotechnol J. 2026 Aug 6;35(1):0196. doi: 10.34133/csbj.0196 (PMC13443017; doi:10.34133/csbj.0196)
Supplement: Supplementary file 1 [file csbj.0196.f1.zip › Supplementary method.docx]

**Supplementary method**

*Concurrent chemoradiation protocol*

Evaluation prior to treatment included a complete physical and pelvic examination, full blood count, renal and hepatic function tests, and serologic screening (HBsAg, VDRL, HIV). The radiotherapy protocol comprised external beam radiation (EBRT) using 6–15 MV photon beams, delivered with either anterior–posterior fields or a four-field box in conventional 2D or 3D conformal RT, and occasionally IMRT. EBRT to the whole pelvis was administered with additional boosts to lymph nodes, parametrium, and pelvic sidewall to a total dose of 50–60 Gy in 20–30 fractions at 2 Gy per fraction. Concurrent weekly platinum-based chemotherapy was given during EBRT (cisplatin 40 mg/m² or carboplatin AUC 2). High-dose-rate intracavitary brachytherapy was then delivered with Iridium-192 remote afterloading, 6.5 Gy per fraction for 3–4 fractions to point A during weeks 3–5 of treatment.

*Nanoparticle Tracking analysis (NTA)*

Membrane particles were eluted from SAX beads using 25 mM Bis Tris Propane, pH 6.5, 1 M NaCl, 0.1% Tween 20. The size and concentration of the particles were then measured using NTA via Nanosight NS300 (Malvern Panalytical, Malvern, UK). Each sample was diluted 1:10–1:20 and recorded at 25 °C, 30 s/video five times. The NTA software determined particle size distribution and concentration (Malvern Panalytical, Malvern, UK).

*Transmission Electron Microscope (TEM)*

Samples were fixed in 2.5% glutaraldehyde in PBS for 30 minutes. A 10 μL aliquot of each EV sample was then applied to a Formvar-coated carbon grid and allowed to adsorb for 30 minutes. Subsequently, 10 μL of 2% uranyl acetate was added for negative staining and incubated in the dark for 10 minutes. Excess stain was removed by blotting the grid at a 45° angle. Imaging was performed using a JEM-1400 TEM (JEOL, Tokyo, Japan).

*Bootstrap resampling of prognostic proteins*

To assess the reproducible selection of the eight-protein prognostic signature, we reran the entire time-varying Cox discovery screen, including multiple-testing correction, on 1,000 patient-level bootstrap resamples. Each resample drew all 42 patients with replacement, retaining each patient's full longitudinal record as a block; repeated draws were assigned distinct cluster identifiers for the robust variance estimator. For each resample, the same stage-adjusted Cox model was refit across the full candidate protein set used in the primary overall survival screen (ANOVA time-significant, q<0.05), and Storey's q-value was recomputed. For each signature protein, we report the percentage of resamples in which it was re-selected as significant (q<0.05) with the same direction of effect as the primary analysis, and a percentile 95% bootstrap CI for the hazard ratio.
