# Supplementary material for: Association between Urinary-Extracellular-Vesicle-Enriched Proteome Dynamics and Oncological Outcomes Following Concurrent Chemoradiation in Locally Advanced Cervical Cancer
Source: Comput Struct Biotechnol J. 2026 Aug 6;35(1):0196. doi: 10.34133/csbj.0196 (PMC13443017; doi:10.34133/csbj.0196)

Supplementary Figure S1. Batch QC: MDS before/after normalisation

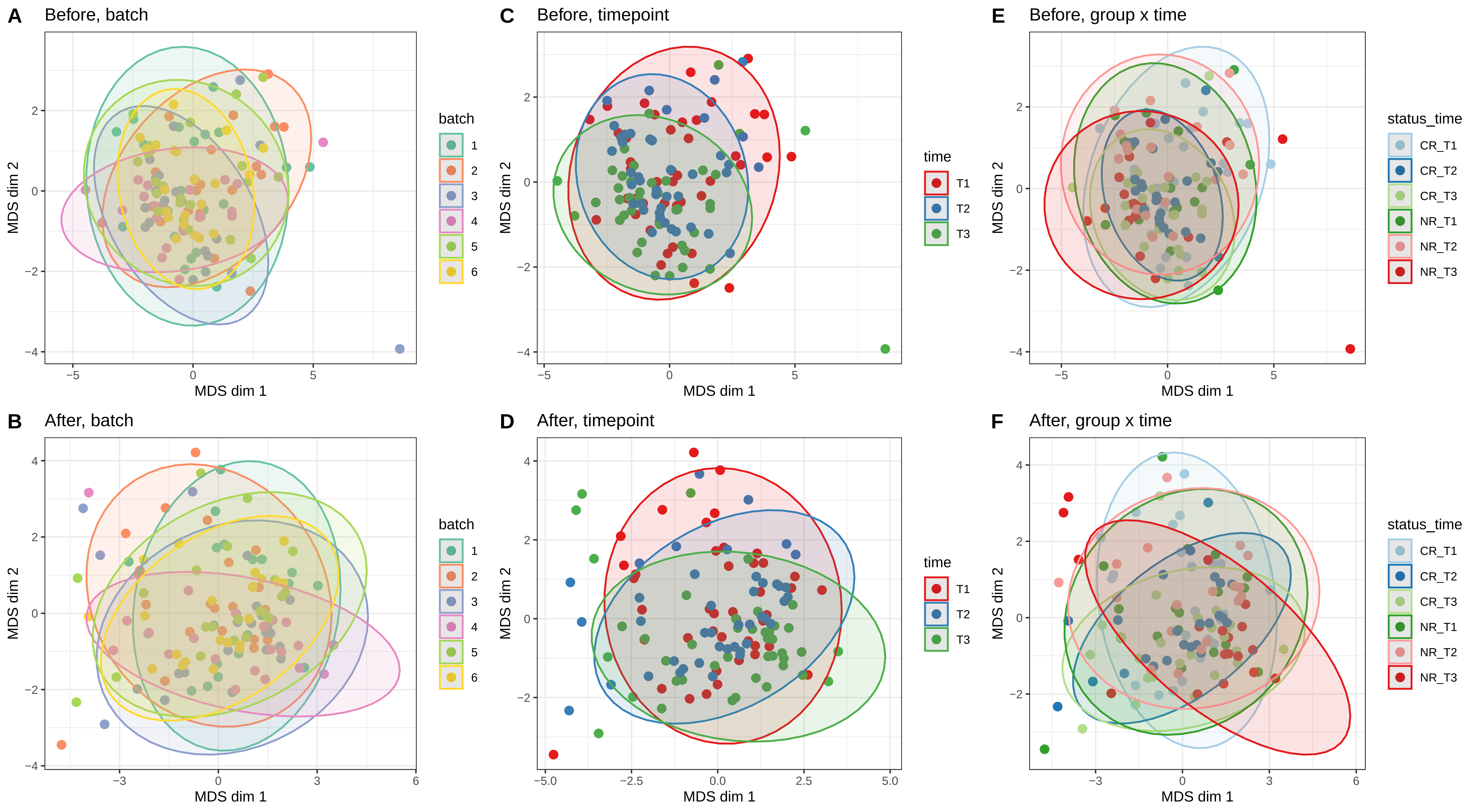

Supplementary Figure S1 (cont). Batch QC: PCA before/after normalisation

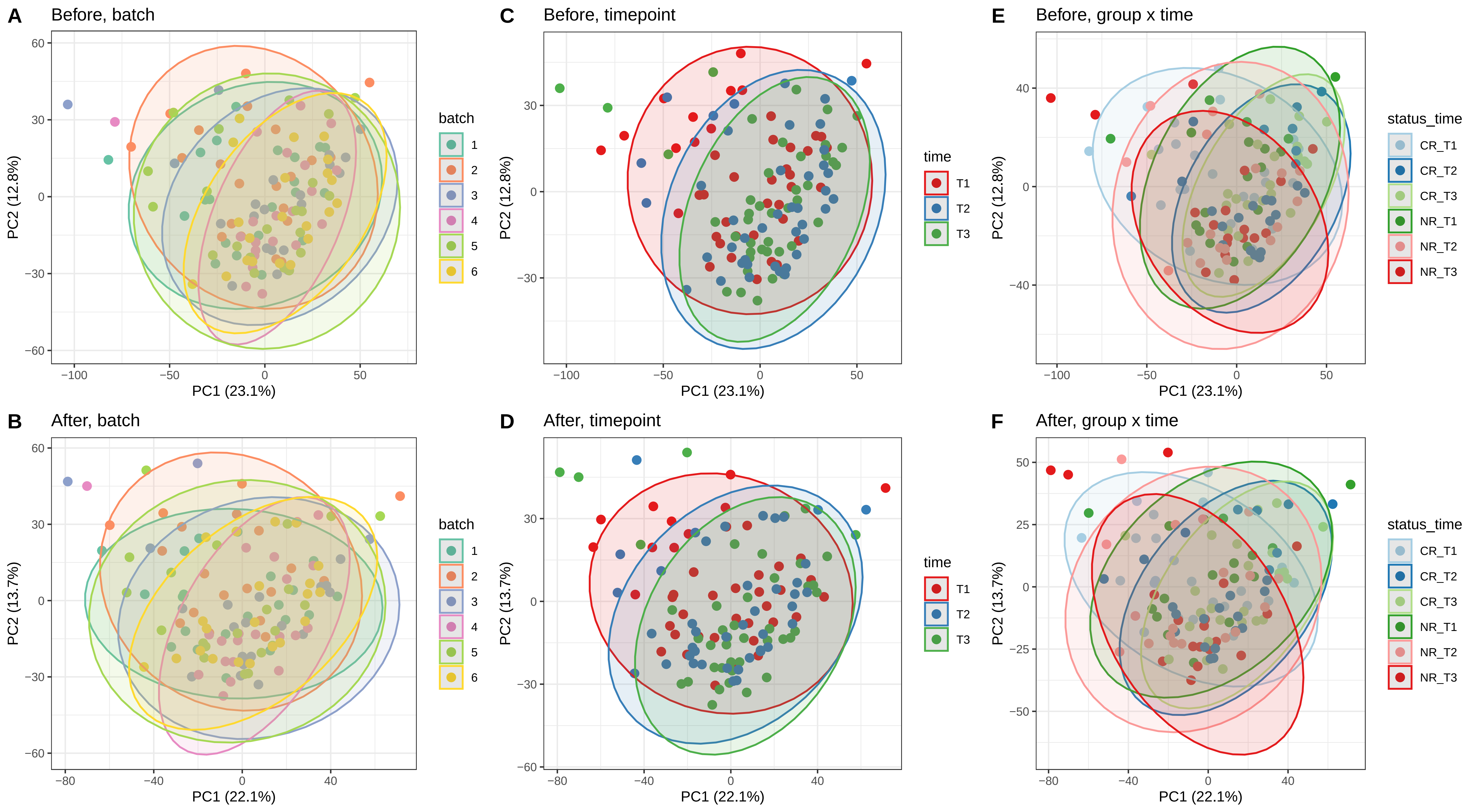

Supplementary Figure S1 (cont). Batch QC: variance partitioning (batch, patient, response status, timepoint). Labels indicate the median % variance explained per variable.

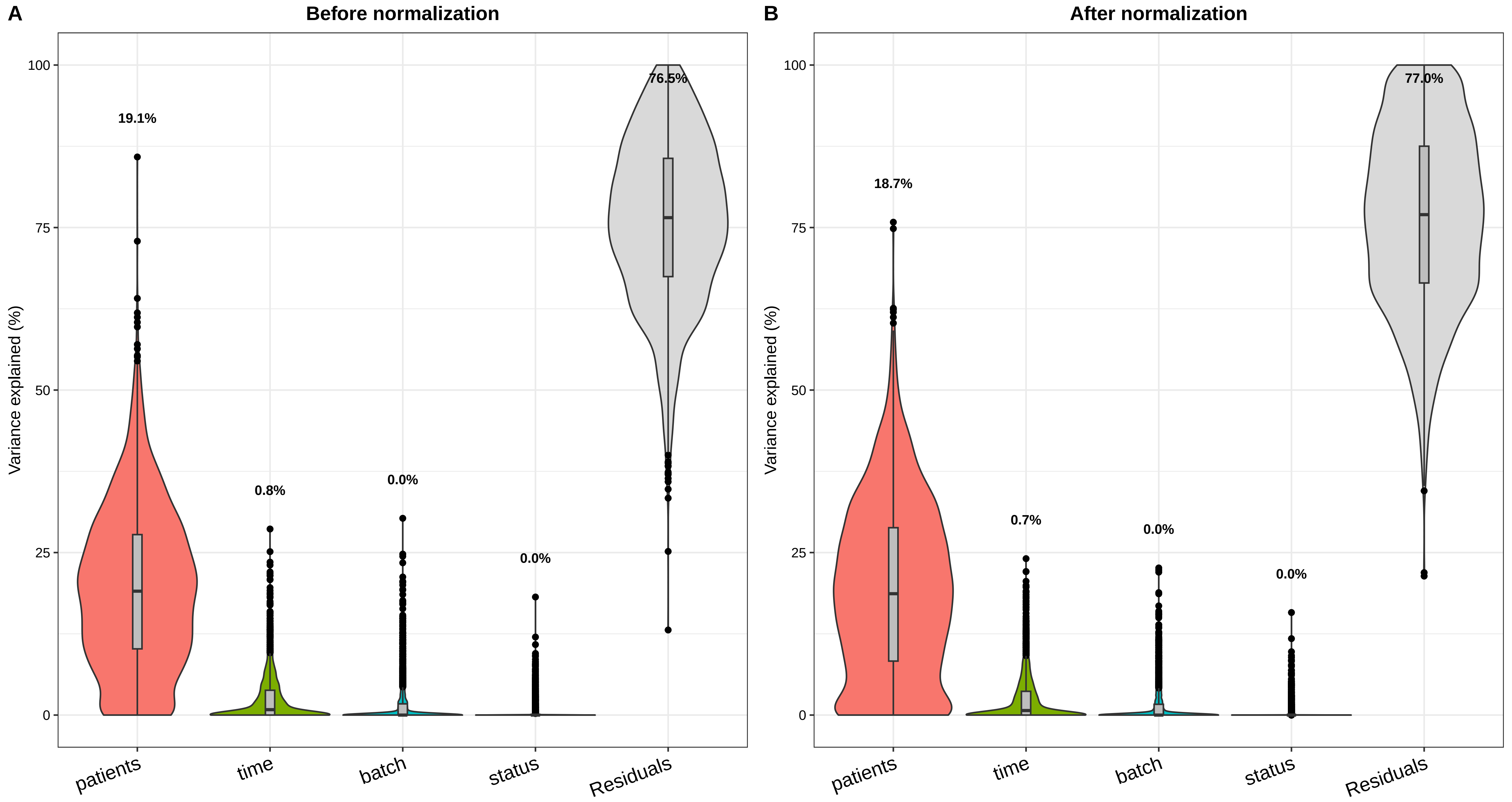

Supplementary Figure S1 (cont). Intensity distribution before/after

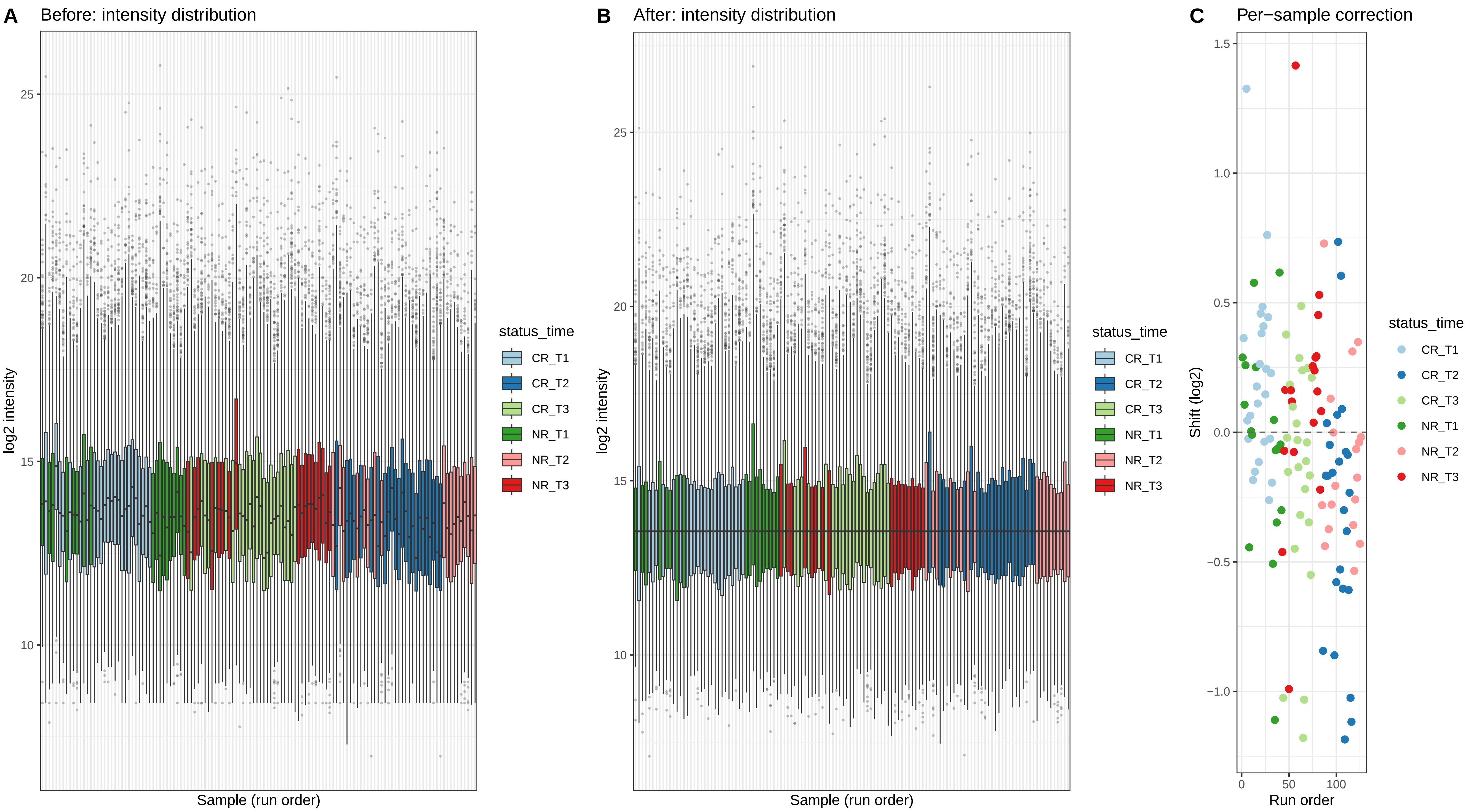

Supplementary Figure S1 (cont). Fold−change distribution before/after

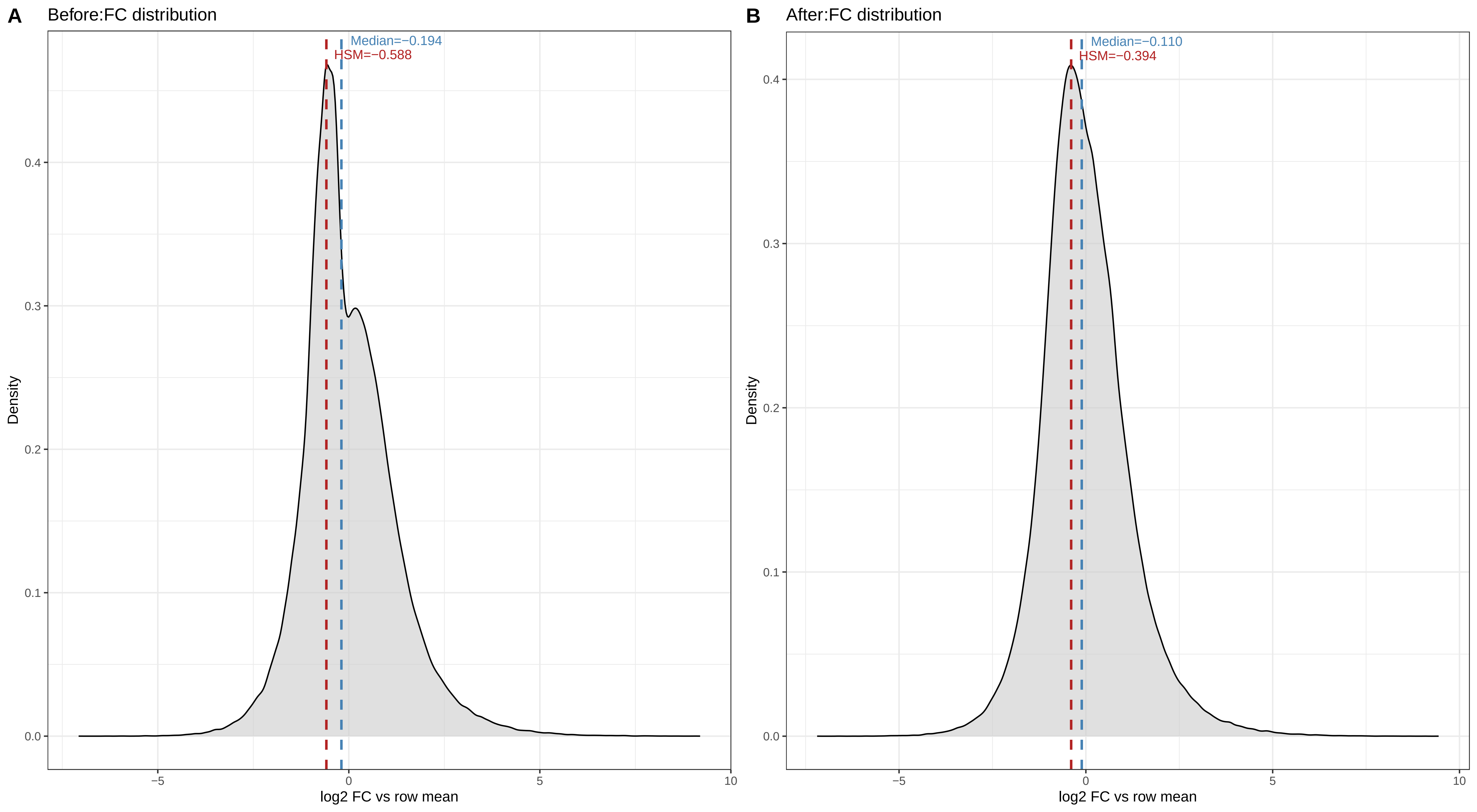

Supplementary Figure S2. Elbow method (within−cluster sum of squares) used to select the optimal number of Mfuzz clusters (k=5)

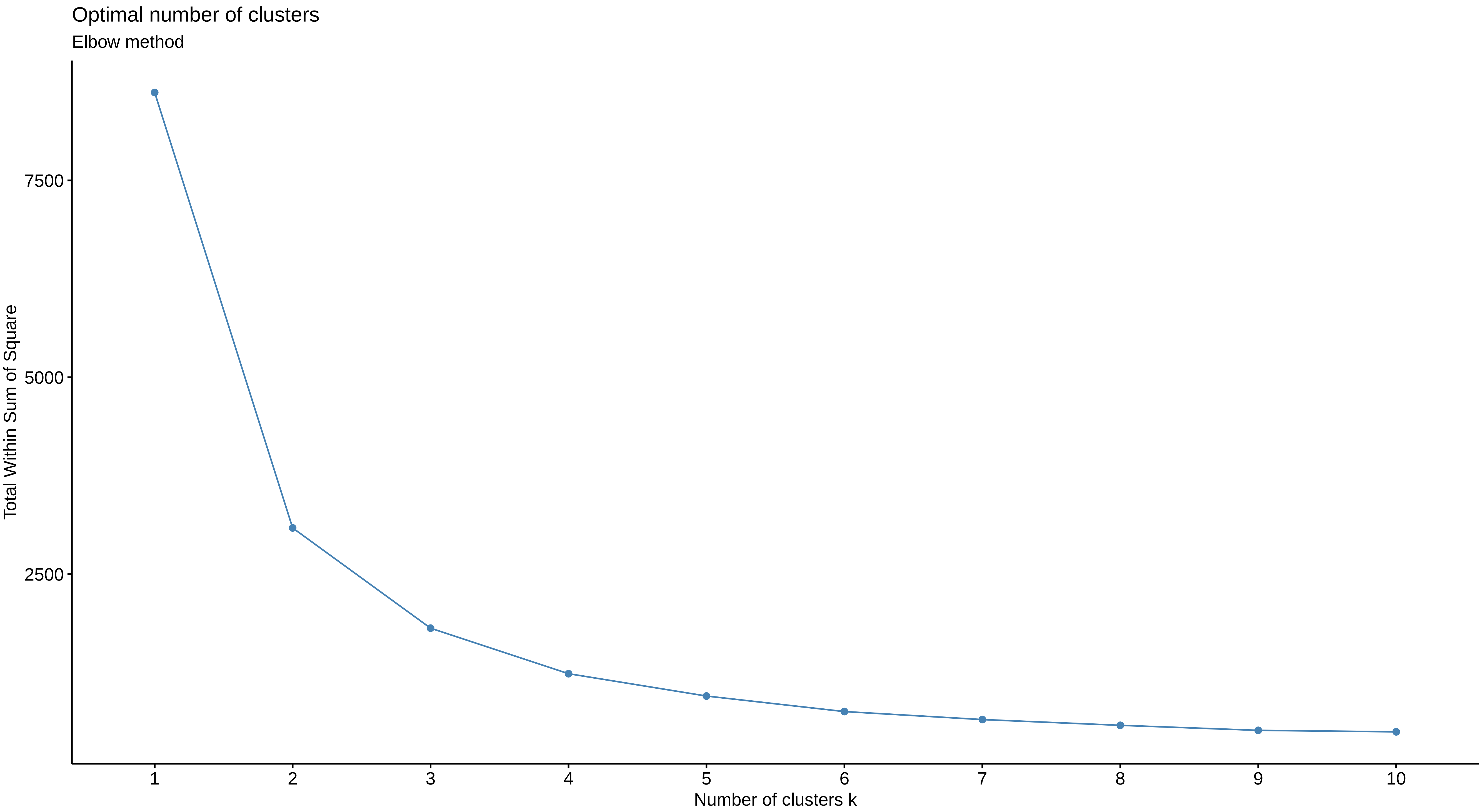

Supplementary Figure S3. Volcano plots: dynamic NR vs CR contrasts

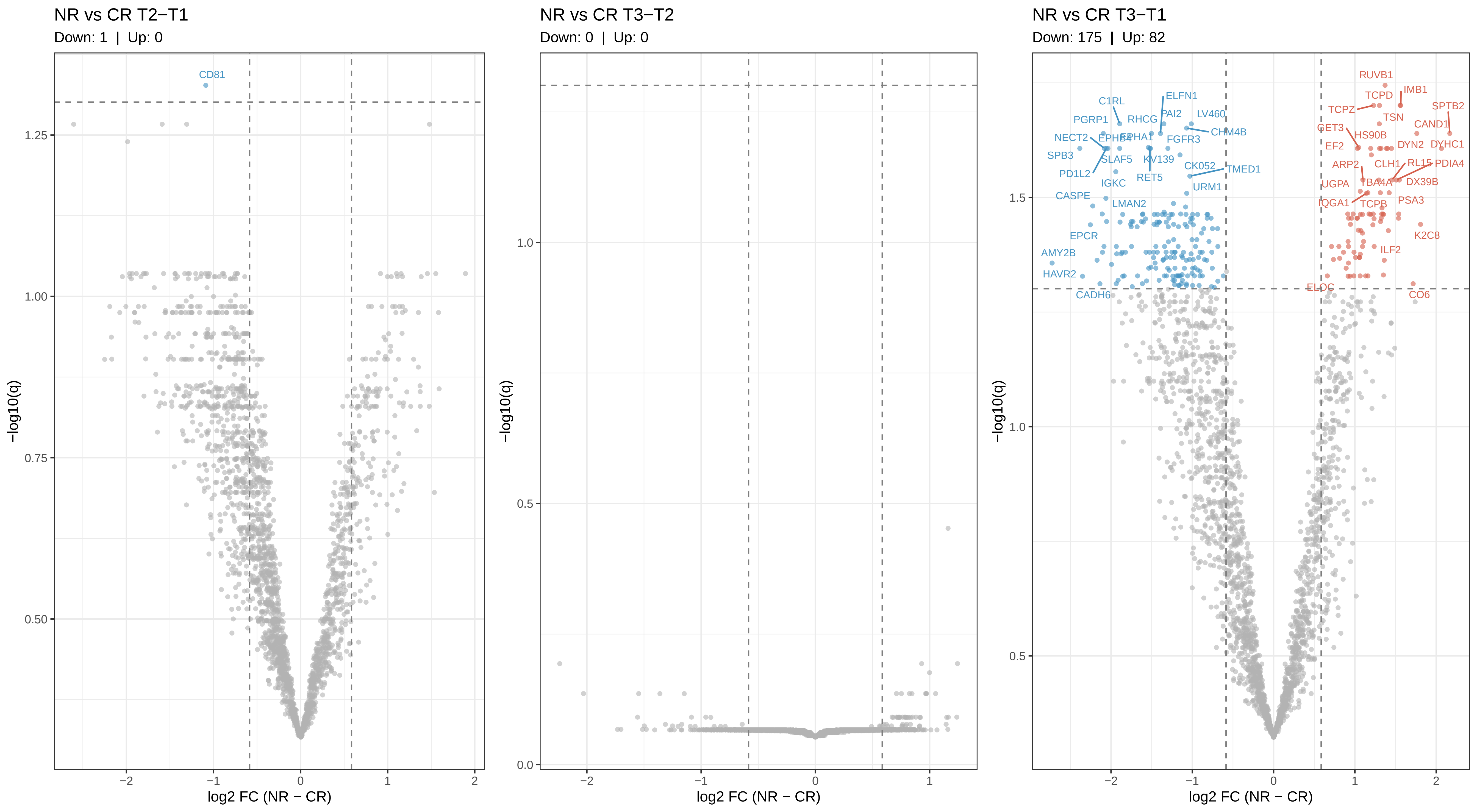

Supplementary Figure S4. Urine OS: all significant proteins (TV Cox)

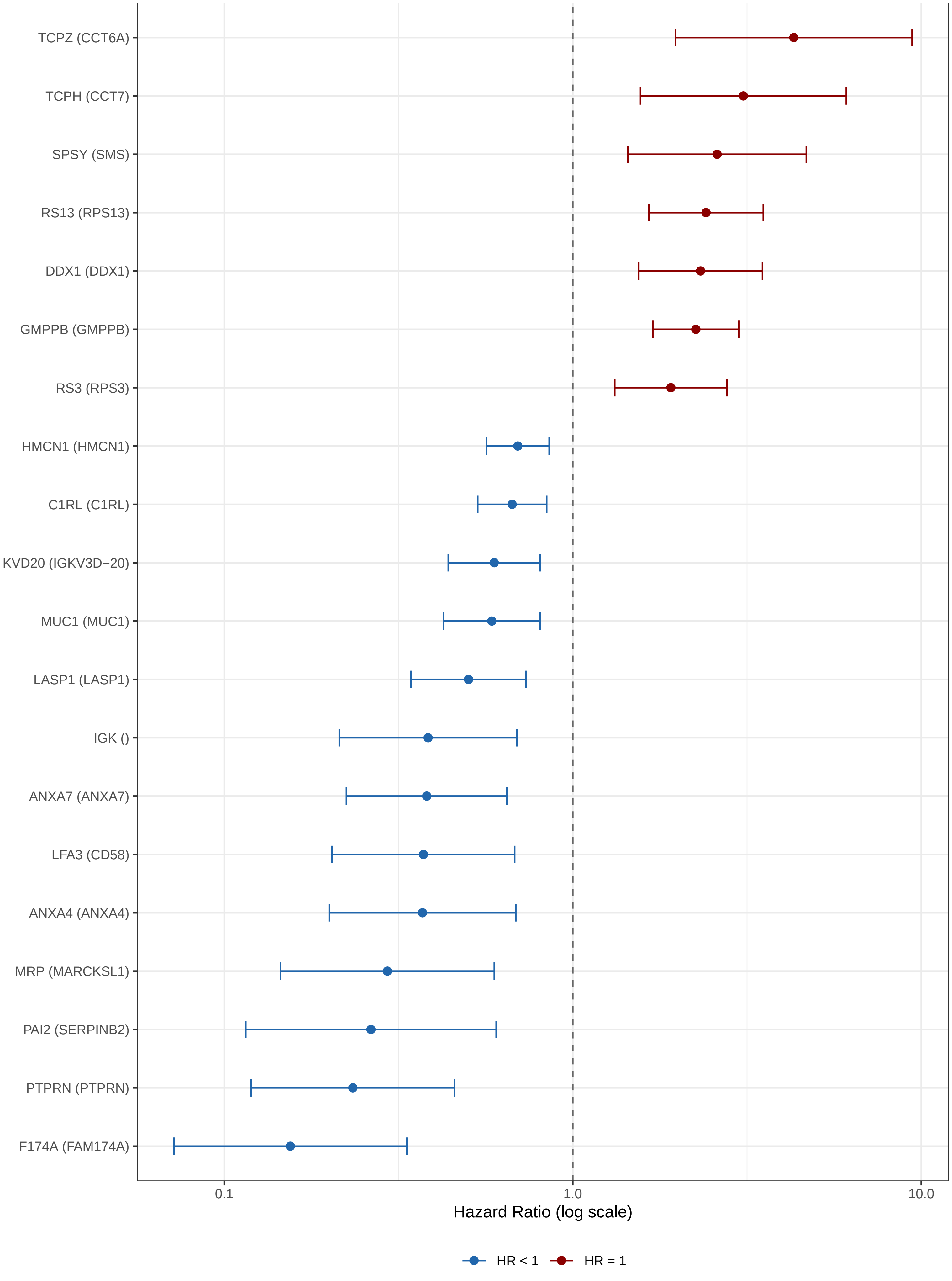

Supplementary Figure S5. Urine OS: sensitivity (all proteins, no ANOVA filter)

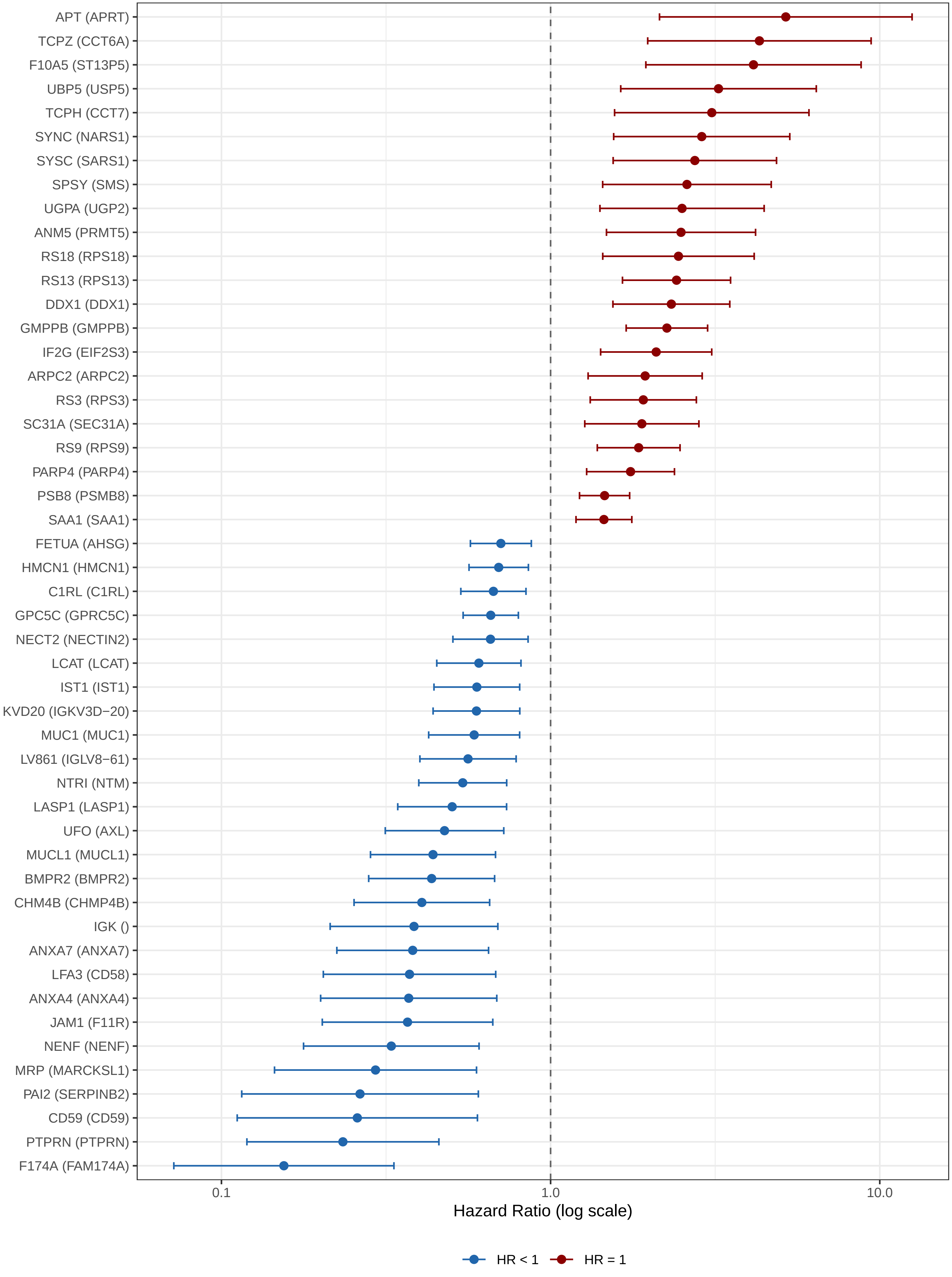

Supplementary Figure S6. Overlap: TV Cox OS (all proteins) and NR vs CR T3−T1 differential proteins

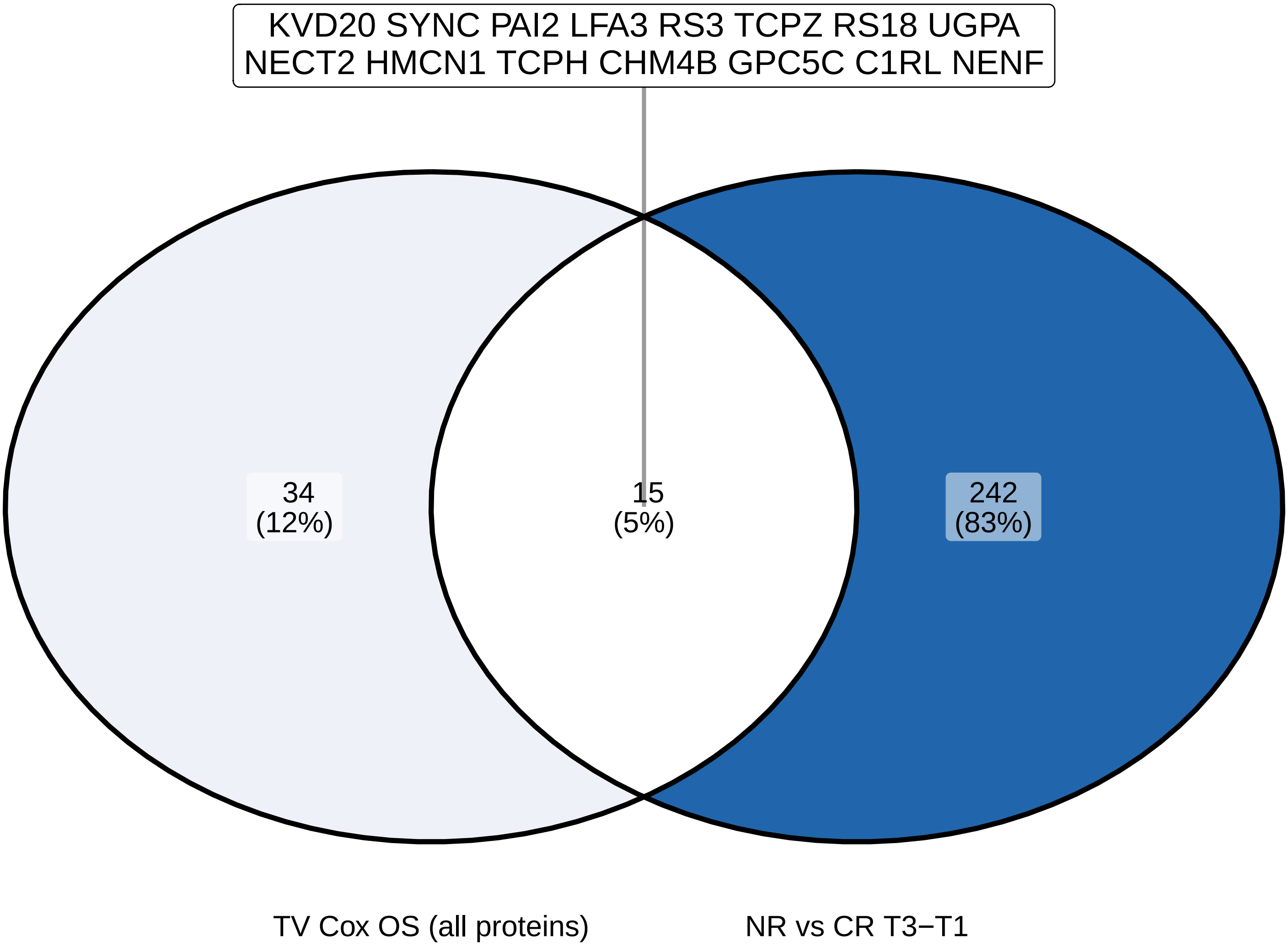

Supplement: Supplementary file 1 [file csbj.0196.f1.zip › Supplementary_figures.pdf]
